# The Role of Mitochondria in Brain Cell Protection from Ischaemia by Differently Prepared Propolis Extracts

**DOI:** 10.3390/antiox9121262

**Published:** 2020-12-12

**Authors:** Zbigniev Balion, Kristina Ramanauskienė, Aistė Jekabsone, Daiva Majienė

**Affiliations:** 1 Laboratory of Pharmaceutical Sciences, Institute of Pharmaceutical Technologies, Lithuanian University of Health Sciences, Sukilėlių ave. 13, LT 50162 Kaunas, Lithuania; zbigniev.balion@lsmuni.lt (Z.B.); aiste.jekabsone@lsmuni.lt (A.J.); 2 Laboratory of Biochemistry, Neuroscience Institute, Lithuanian University of Health Sciences, Eivenių str. 4, LT-50161 Kaunas, Lithuania; 3 Department of Clinical Pharmacy, Lithuanian University of Health Sciences, Sukilėlių ave. 13, LT 50162 Kaunas, Lithuania; kristina.ramanauskiene@lsmuni.lt; 4 Laboratory of Preclinical Drug Investigation, Institute of Cardiology, Lithuanian University of Health Sciences, Sukilėlių ave. 13, LT-50162 Kaunas, Lithuania; 5 Department of Drug Technology and Social Pharmacy, Lithuanian University of Health Sciences, Sukilėlių ave. 13, LT 50162 Kaunas, Lithuania

**Keywords:** hypoxia, neurons, astrocytes, microglia, mitochondrial superoxide, lactate dehydrogenase, oxygen consumption rate, aqueous propolis extract, polyethylene-aqueous propolis extract, ethanolic propolis extract

## Abstract

Mitochondria are both the primary targets and mediators of ischaemic damage in brain cells. Insufficient oxygen causes reactive oxygen species that damage the mitochondria, leading to the loss of functionality and viability of highly energy-demanding neurons. We have recently found that aqueous (AqEP), polyethylene glycol-aqueous (Pg-AqEP) and ethanolic propolis extracts (EEP) can modulate mitochondria and ROS production in C6 cells of astrocytic origin. The aim of this study was to investigate the effect of the extracts on viability, mitochondrial efficiency and superoxide generation, and inflammatory cytokine release in primary rat cerebellar neuronal-glial cell cultures affected by ischaemia (mimicked by hypoxia +/− deoxyglucose). AqEP and Pg-AqEP (15–60 µg/mL of phenolic compounds, or PC) significantly increased neuronal viability in ischaemia-treated cultures, and this was accompanied by a reduction in mitochondrial superoxide levels. Less extended protection against ischaemia-induced superoxide production and death was exhibited by 2 to 4 µg/mL of PC EEP. Both Pg-AqEP and Ag-EP (but not EEP) significantly protected the cultures from hypoxia-induced elevation of TNF-α, IL-1β and IL-6. Only Pg-AqEP (but not AqEP or EEP) prevented hypoxia-induced loss of the mitochondrial basal and ATP-coupled respiration rate, and significantly increased the mitochondrial respiratory capacity. Summarising, the study revealed that hydrophilic propolis extracts might protect brain cells against ischaemic injury by decreasing the level of mitochondrial superoxide and preventing inflammatory cytokines, and, in the case of Pg-AqEP, by protecting mitochondrial function.

## 1. Introduction

Ischaemia causes impairment of oxygen and nutrient supply to the affected region, and thereby induces tissue and organ damage. The brain consumes about 20% of the total body oxygen; thus, a lack of oxygen is extremely dangerous to neural cells. According to the WHO, ischaemic brain diseases are among the leading causes of mortality and disability in Western countries. Annually, about 16 million stroke cases occur in the world, causing a total of 5.7 million deaths [1].

Several mechanisms play a role in the pathophysiology of ischemic stroke, such as oxidative stress, inflammation and cell death. Upon cerebral ischaemia/reperfusion, a cascade of pathophysiological events is initiated in the infarct area, including infiltration of peripheral inflammatory cells, activation of microglia cells, excessive production of inflammatory mediators and matrix metalloproteases [2]. There are also studies indicating that one of the most important factors determining the level of damage is the amount of adenosine triphosphate (ATP) in the cells [3]. About 80–90% of cellular ATP is produced in mitochondria during the process of oxidative phosphorylation. The intensity of the process directly depends on the oxygen level, and a significant drop in oxygen progressively decreases the ATP concentration [4]. Ischaemia also induces development of structural mitochondrial changes, leading to the decreased activity of oxidative phosphorylation [5]. These mitochondrial events lead to both cell de-energization and generation of highly damaging reactive oxygen species (ROS), such as hydroxyl radicals and superoxide radicals, that might further damage mitochondria and other vitally important intracellular structures and contribute to the ischaemia-induced failure of neural tissue [6,7]. A substantial increase in ROS promotes the irreversible opening of the mitochondrial permeability transition pore, and after this, the organelle is completely switched out from the energy production process [8,9]. All these above-listed events are the main players inducing inflammatory responses and determining neural cell fate during ischaemia/reperfusion, leading to apoptotic, autophagic or necrotic death [10,11,12]. In spite of the variety of available therapeutic options, high prevalence rates of stroke are still recorded due to the low efficacy of neuroprotective drugs. Therefore, new alternative therapeutic strategies are urgently required to reduce the incidence and to treat the consequences of stroke.

The use of natural herbal medicines and phytonutrients has continuously grown during recent years. The products may possess significant therapeutical efficiency and a far lower toxicity compared to synthetic drugs. Thus, the use of natural compounds has no or very little side effects, even after prolonged administration.

One of the plant-derived natural materials with very strong and wide biological activities is a bee product propolis. It is comprised of 80 to 300 active compounds, depending on the plants growing around the beehives. Propolis is also well-known in folk medicine and is effectively used for treatment of various human diseases [13]. The amount of research done on propolis during the last decades demonstrates a vast spectrum of its biological activity, such as antiseptic, anti-inflammatory, antioxidant, antibacterial, antifungal, antineoplastic, hepatoprotective, cardioprotective and immunomodulatory. [14,15]. The therapeutical potential of propolis is determined by the variety of biologically active substances, such as phenolic compounds, the majority of which are flavonoids, aromatic acids and their esters, etheric oils, microelements, vitamins and amino acids. In several studies, flavonoids have demonstrated an ability to prevent myocardial ischaemia/reperfusion-induced injuries [16,17]. The effect is exerted via antioxidative, vasodilative, anti-inflammatory and anti-aggregative activities of the substances. Polyphenols and flavonoids are powerful antioxidants due to the hydrogen-donating ability of their hydroxyl groups as well as their ability to donate electrons to arrest the production of free radicals resulting from oxidative stress [18]. Some of the chemical components of propolis, such as galangin, caffeic acid phenethyl ester, baccharin, p-Coumaric acid and Artepillin C, are reported to have potential neuroprotective activity [19,20]. Moreover, propolis components protected the cultured neuronal cells RGC 5 and PC12 from oxygen and glucose deprivation-induced damage via modulation of apoptosis and antioxidant activity-related genes [21]. Furthermore, the neuroprotective effect of propolis was demonstrated in a rat focal cerebral ischaemia model made by middle cerebral artery occlusion; the activity was likely to be mediated through antioxidant effect and upregulation of TGF-b1 [22]. Despite these promising findings, the mechanism of propolis upon ischaemia-induced brain injuries has not been thoroughly investigated.

The majority of propolis constituents are lipophilic and are easily dissolved in polar solvents (ethanol and methanol). That is why propolis is commonly used as a liquid ethanolic extract [13]. However, ethanol and similar polar solvents are irritative to living tissues; for this reason, the administration capacity and pharmacological applicability of the extracts is limited. Water extracts are the most tissue-friendly, however, in such preparations, the levels of biologically active compounds were found to be 10–20 times lower compared to the ethanolic extracts [23]. To increase the applicability of propolis, an aqueous and solvent complex, based on an aqueous solution with 20% polyethylene glycol, has been produced. Thus, by adding co-solvents to water and by modifying the production conditions, higher levels of active compounds are achieved. However, different extraction solvents can extract different biologically active compounds, resulting in distinct biological effects. Phenolic acids and aldehydes make up 40–42% of all extracted and identified compounds in aqueous (AqEP) and polyethylene glycol–aqueous (Pg-AqEP) propolis extract, and only 16% in ethanolic propolis extract (EEP) [23]. All preparations revealed similar antioxidant activity in cell-free culture medium, but in a C6 astrocytoma cell culture, Pg-AqEP and EEP demonstrated better mitochondrial superoxide- and total intracellular ROS-decreasing properties [24]. In addition, Pg-AqEP had a concentration-dependent, mitochondria-uncoupling effect, the property that might prevent irreversible opening of the mitochondrial permeability transition pore [25]. These ROS and mitochondria-modulating properties indicate that propolis extracts might have impact on mitochondrial and neural cell health during ischaemia–reperfusion injury. Therefore, in this study, we have examined how AqEP, Pg-AqEP and EEP affect the viability, mitochondrial functions, mitochondrial superoxide production and inflammatory cytokine release in primary neuronal and glial cell cultures affected by ischaemia that was mimicked by hypoxia in the presence of deoxyglucose.

## 2. Materials and Methods

### 2.1. Propolis Extracts: Preparation and Chemical Analysis

Prior to analysis, propolis samples were kept at room temperature in the dark. Propolis extracts were prepared as described in [23]. Briefly, crude propolis was grounded into powder and macerated in different solvents (water, 20% PEG/water and 70% ethanol) by shaking. Extraction time—5 h at room temperature. The propolis sample-to-solvent ratio was 1:10 (w/v). After extraction, extracts of propolis were filtered through paper filter. The solutions were clear, yellow liquids and remained stable when stored up to one week in the dark at 4 °C [23]. Analysis of the extracts by High-Performance Liquid Chromatography was done using an Agilent 1260 Infinity capillary LC (Agilent Technologies, Inc., Santa Clara, CA, USA) [24]. The total amount of phenolic acids in AqEP, Pg-AqEP and EEP were 514.7 ± 46.0, 879.6 ± 29.8 and 3404.7 ± 124.8 μg/mL, respectively; the total amount of phenolic compounds (PC) were 1207.9 ± 27.6, 2149.5 ± 16.1 and 20,791.3 ± 2320.9 μg/mL, respectively [24].

### 2.2. Neuronal-Glial Cell Culture

A rat cerebellar neuronal-glial cell culture from postnatal 5–7-day-old Wistar rats was prepared as described in [26]. Briefly, the cerebella were isolated, minced and triturated in Versene solution (1:5000; Gibco, Thermo Fisher Scientific) until a single-cell suspension was obtained from the tissue. The suspension was centrifuged at 270× *g* for 5 min and resuspended in DMEM with Glutamax (Thermo Fisher Scientific) supplemented with 5% horse serum, 5% foetal calf serum, 38 mM glucose, 25 mM KCl and antibiotic–antimycotic (Thermo Fisher Scientific). The cells were plated at a density of 0.25 × 10^6^ cells/cm^2^ in 96-well plates (VWR) coated with 0.0001% poly-L-lysine and kept at 37 °C in a humidified incubator containing 5% CO_2_. The cultures were subjected to treatments after 7 days in vitro.

### 2.3. Simulated Ischaemia Model and Cell Culture Treatments

After a week in vitro, cell cultures were subjected to 24 h simulated ischaemia by placing them in a humidified 37 °C chamber perfused by a gas mixture of 2% O_2_/5% CO_2_/93% N_2_ to induce hypoxia. For inhibition of glycolysis, 10 mM D-deoxyglucose (DOG) was added to the cell culture medium. To investigate the effect of the propolis extracts on the level of hypoxia + DOG-induced necrosis and mitochondrial superoxide production, the cultures were treated with 7.5–60 µg/mL PC of AqEP, 10–60 µg/mL PC of Pg-AqEP and 2–6 µg/mL PC of EEP. In parallel, the hypoxic control without DOG, and the extracts without DOG were tested; also, controls with the same amount of solvents without the propolis extracts were prepared. The effects of all the above-listed treatments were also tested in normoxic conditions over the same 24 h period. For investigation of superoxide production and mitochondrial functions, hypoxic treatments were performed without DOG.

### 2.4. Identification of Neuronal, Astrocyte and Microglial Cells, Viable and Necrotic Nuclei

After the treatments, viability staining of nuclei was performed by adding propidium iodide (PI, 3 µg/mL) and Hoechst 33342 (6 µg/mL) to the incubation media and allowing the dyes to penetrate into cells, incubating for 5 min at 37 °C. Neurons in cell cultures were identified by the synaptosomal-associated protein, 25 kDa (SNAP-25, RRID:AB_2192212), and astrocytes by the glial fibrillary acidic protein (GFAP, RRID:AB_10984338). For that, cultures were fixed in 4% paraformaldehyde in PBS for 15 min at room temperature, blocked and permeabilised with 0.4% Triton X-100 and 20% BSA for 16 h at 4 °C, and incubated for 3 h with primary antibodies: rabbit polyclonal anti-SNAP25 (Thermo Fisher Scientific, 10 µg/mL) or mouse monoclonal anti-GFAP (Thermo Fisher Scientific, 4 µg/mL) in PBS at room temperature. Then the cultures were treated with secondary antibodies AlexaFluor555-conjugated goat anti-mouse IgG or AlexaFluor555-conjugated goat anti-rabbit IgG (from Thermo Fisher Scientific and diluted in PBS 1:200 with 5% BSA) for 2 h at room temperature. Microglial cells were affinity-stained with isolectin IB4 from *Griffonia simplificolia* conjugated with AlexaFluor488 (20 ng/mL, 1 h at room temperature). The stained cells in the cultures were visualized under a fluorescent microscope (Olympus IX71, Olympus Corporation, Tokyo, Japan), with the ×20 objective, and the images were taken by the camera 01-Exi-AQA-R-F-M-14-C (QImaging, Surrey, BC, Canada) and ImageProExpress software. Necrotic versus viable cells were identified under the DAPI filter set with an excitation band pass 352–402 nm and long pass emission filter transmitting the light waves longer than 410 nm. Hoechst33342-only-positive nuclei exhibiting blue fluorescence were considered viable, and Hoechst3334-plus-PI-positive nuclei fluorescing magenta were identified as necrotic. Small nuclei with condensed chromatin, visible as very bright blue, were considered apoptotic. AlexaFluor555 were excited and visualized under a TxRed band pass filter set (540–550 nm for excitation and 575–625 for emission). The dye is not excited under DAPI filter excitation light; thus, it does not interfere with the PI staining under this filter set. Microglial cells (AlexaFluor488 fluorescence) were visualized under the FITC band pass filter set (467–498 nm for excitation and 513–555 nm for emission). Cell cultures were also visualized under a brightfield microscope. The image analysis was performed using ImageJ software.

### 2.5. Measurement of Lactate Dehydrogenase Activity

Lactate dehydrogenase (LDH) activity was measured using the colorimetric Cytotoxicity Detection Kit PLUS LDH (Sigma-Aldrich). The standard protocol assays were performed according to the manufacturer’s instructions. The data were expressed as the percentage of total cellular LDH content.

### 2.6. Caspase-3 Activity Assessment

Caspase-3 activity in cell lysates after treatments was measured by means of the Caspase 3 Assay Kit (Merck), according to the manufacturer’s protocol. Briefly, the activity of caspase-3 was evaluated according to Ac-DEVD-7-amido-4-methylcoumarin cleavage and subsequent increase in 7-amido-4-methylcoumarin (AMC) fluorescence, detecting the signal change in a fluorometric plate reader (Ascent Fluoroskan, Thermo Fisher Scientific, Inc. Waltham, MA, USA; λ_ex_ = 360 nm, λ_em_ = 460 nm). For the positive control of the assay (additional to the active caspase-3 test provided by the manufacturer), 100 nM staurosporine (Merck) was applied for 3 h. The data are presented as averages ± standard error of the caspase inhibitor Z-DEVD-CHO-sensitive increase in AMC concentration normalized to mg of cellular protein (evaluated by Braford assay) averages ± standard error.

### 2.7. Evaluation of Extracellular Hydrogen Peroxide

The H_2_O_2_ amount was determined fluorometrically using 10-acetyl-3,7-dihydroxyphenoxazine (Amplex^®^ Red, Thermo Fisher Scientific). In combination with horseradish peroxidase, this dye reacts with H_2_O_2_ in a 1:1 stoichiometry to produce the red-fluorescent resorufin. After hypoxia plus DOG treatments with or without propolis extracts, the cells were washed thrice with Hank’s Balanced Salt Solution (HBSS) and subjected to Amplex^®^ Red (5 μM) in the presence of horseradish peroxidase (HRP; 2 U/mL). The fluorescence intensity of the resulted resorufin was detected by a fluorometer (Ascent Fluoroskan, Thermo Fisher Scientific, Inc. Waltham, MA USA) at excitation and emission wavelengths of 544 and 590 nm, respectively. The measurements were made at time point “0” and after 30 min. The difference in fluorescence intensity over that period was used to calculate the H_2_O_2_ concentration according to the calibration with known amounts of H_2_O_2_. For the control, the level of H_2_O_2_ was determined in HBSS containing the appropriate amount of the solvents only.

### 2.8. Measurement of Intracellular Reactive Oxygen and Nitrogen Species

Generation of reactive oxygen and nitrogen species in neuronal-glial cells after hypoxia plus DOG treatment in the presence of different propolis extracts were measured as described in [27], with small modifications. After treatments, the cells were washed thrice with 37 °C HBSS with Ca^2+^ and Mg^2+^ (HBSS/Ca/Mg, Thermo Fisher Scientific) and incubated with 10 μM 2′,7′-dichlorofluorescin diacetate (DCFDA, Cat. No. D6883, Sigma-Aldrich) in HBSS/Ca/Mg in the dark at 37 °C for 30 min. After incubation, the excessive DCFDA was washed away with HBSS/Ca/Mg and the fluorescence was read in a multifunctional plate reader Infinite 200 Pro M Nano Plex (Tecan, Männedorf, Switzerland) at excitation/emission wavelengths 485 nm/535 nm. The readings were performed immediately after loading of the dye and after 0.5, 1, 1.5 and 2 h.

### 2.9. Evaluation of Mitochondrial Superoxide

For evaluation of mitochondrial superoxide, the cells after treatments were washed thrice with HBSS and incubated with 2 µM MitoSox^TM^ Red (Cat. No. M36008, Thermo Fisher Scientific) in HBSS at 37 °C in the dark for 15 min. The excess dye was removed by an additional three washes with HBSS, and the images were taken by fluorescent microscopy (Zeiss Axio Observer.Z1). The fluorescence intensity in the images was evaluated by ImageJ software.

### 2.10. Evaluation of Mitochondrial Functions

Assessment of mitochondrial and glycolytic functionality was performed by a Seahorse XFp Analyser (Agilent Technologies, Santa Clara, CA, USA) using Seahorse XFp Cell Mito Stress Test Kit (Agilent Technologies) and Seahorse XFp Real-Time ATP Rate Assay Kit (Agilent Technologies), according to manufacturer’s instructions. The cells were seeded into the Agilent Seahorse XFp well plates at a density of 500,000 cells/cm^2^ and grown, as described in Section 2.1., in an incubator for 7 days. Then, the cells were 24-h treated in a hypoxic chamber, as described in Section 2.2. (the concentrations of AqEP, Pg-AqEP and EEP were 60 µg/mL of PC, 60 µg/mL of PC and 2 µg/mL of PC, respectively), with or without the propolis extracts. One hour before the measurement, the medium was replaced with Seahorse XF Assay Medium supplemented with 2 mM L-glutamine, 1 mM sodium pyruvate and 25 mM glucose, and the cells were placed in a non-CO_2_ incubator. Just before the measurement, the medium was changed again to the fresh Assay Medium with the same supplements. The final inhibitor concentrations in the wells were 1.5 μM oligomycin, 1 μM carbonyl cyanide-4-phenylhydrazone (FCCP), 0.5 μM antimycin A and 0.5 μM rotenone. The oxygen consumption rate (OCR) and extracellular acidification rate (ECAR) were normalized to the total cellular protein content, determined directly in the plate immediately after each experimental run by Bradford assay. Optical density after reaction with the Bradford reagent (Cat. No. B6916, Merck) was assessed by a multifunctional plate reader (Infinite 200 Pro M Nano Plex, Tecan, Männedorf, Switzerland). The data were analysed and single-run reports were generated by Wave software (Agilent Technologies), and graphical images of the summary data were created by SigmaPlot vs.13 (Systat Software Inc., Slough, UK).

### 2.11. Measurement of Mitochondrial Inner Membrane Potential

Mitochondrial inner membrane potential in cells after treatments was assessed by a TMRE Mitochondrial Membrane Potential Assay Kit (Cayman Chemical Company), according to the protocol provided by the manufacturer. The assay is based on accumulation of a cell-permeable, cationic dye tetramethylrhodamine ethyl ester (TMRE) in the mitochondrial matrix, according to the value of the mitochondrial membrane potential (Δψ). Briefly, the cells were incubated with 200 nM TMRE at 37 °C for 30 min, washed with the Assay Buffer, and then the fluorescence intensity of the incorporated dye was measured in a multifunctional plate reader (Infinite 200 Pro M Nano Plex, Tecan, Männedorf, Switzerland; excitation/emission = 530/580 nm). For assay sensitivity control, 10 μM FCCP was used to disrupt the mitochondrial membrane potential.

### 2.12. Evaluation of Inflammatory Cytokine Concentration

The cell culture medium collected after hypoxia plus DOG treatments with and without propolis extracts was assayed for the cytokines TNF-α, IL-1β and interleukin-6 (IL-6) by ELISA kits (from Abcam), following the manufacturer’s protocols.

### 2.13. Statistical Analysis

Results are presented as the means of 3–7 experiments (performed in three technical replicates) ± standard error. Statistical analysis was performed by one-way analysis of variance (ANOVA), followed by Dunnett’s post-test using the software package SigmaPlot version 13.0 (Systat Software Inc., Slough, UK). A *p*-value < 0.05 was taken as the level of significance.

## 3. Results

### 3.1. The Effect of Propolis Extracts on Ischaemia-Treated Cerebellar Cell Viability

**Figure 1:**
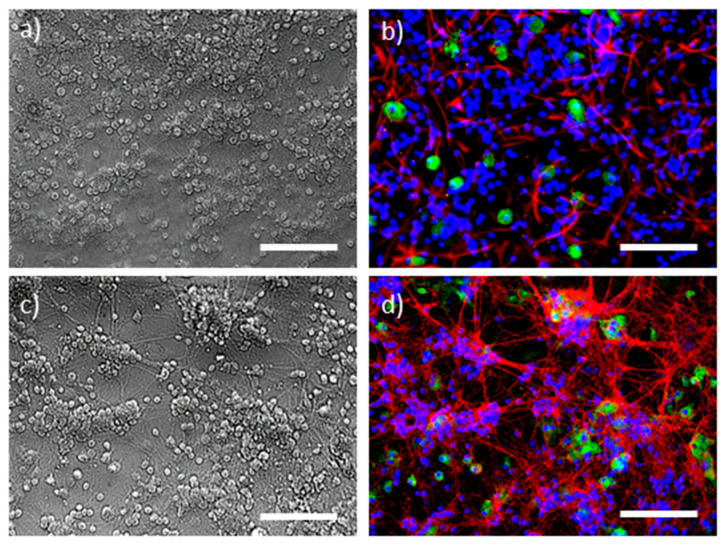
Brightfield (**a**,**c**) and fluorescent (**b**,**d**) images of cerebellar neuronal-glial cell cultures. Image (**b**) shows the same cells as in (**a**), and image (**c**) the same cells as in (**d**). In both fluorescent images, all nuclei were stained blue with Hoechst33342, and the microglia are stained green with isolectin GS-IB4. In (**b**), astrocytes (red) are immunolabelled for glial fibrillary acidic protein (GFAP), and in (**d**) the neurons (red) are immunolabelled for synaptosomal-associated protein, 25 kDa (SNAP-25). Scale bars are 100 μm.

**Figure 2:**
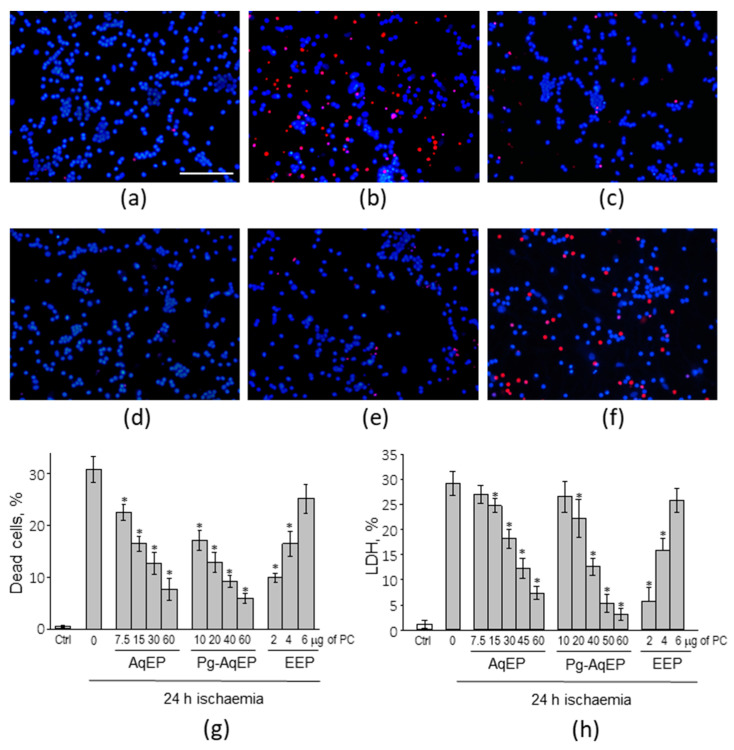
The effect of the propolis extracts on simulated ischaemia (hypoxia plus DOG)-treated cerebellar cell viability. (**a**–**f**) Representative images of the double nuclear viability staining with Hoechst33342 and propidium iodide (PI). Blue nuclei indicate the cells have an intact nuclear membrane and are considered viable. Red, or PI-positive nuclei, are considered necrotic because of lost membrane integrity. (**a**)—image of the control sample; (**b**–**f**)—images of the hypoxia and deoxyglucose-treated samples, (**b**)—without additional treatments, (**c**)—together with 60 μg/mL phenolic compounds AqEP, (**d**) – together with 60 μg/mL phenolic compounds Pg-AqEP, (**e**)—with 2 μg/mL phenolic compounds EEP, and (**f**)—with 6 μg/mL phenolic compounds EEP. Scale bar is 100 μm. (**g**) The percentage of dead cells per microscope image. Data are presented as an average ± SE of 3 experiments; 10 images of each sample were evaluated in each experimental set. (**h**) The percentage of total cellular LDH released to the cell culture medium during the treatment. Data are presented as an average ± SE of 3 experiments performed in triplicate. For both (**g**) and (**h**), *—statistically significant difference compared with the hypoxic control (0), *p* < 0.05; all hypoxic samples were significantly different compared to the normoxic control, *p* < 0.001.

To simulate ischaemia, a primary cerebellar mixed neuronal-glial cell culture (Figure 1), comprising 81 ± 4% granule neurons, 14 ± 3% astrocytes and 6 ± 2% microglial cells, as identified in Balion et al. (2020) [26], was treated with 10 mM deoxyglucose (DOG) and subjected to hypoxic conditions for 24 h. Simulated ischaemia caused 31% of cells with necrotic propidium iodide-positive nuclei (Figure 2b,g). The treatment of cells with differently prepared propolis extracts showed a dose-dependent increase in cell viability. Samples incubated with small concentrations AqEP and Pg-AqEP (up to 7.5 and 10.0 μg of PC, respectively) did not significantly change the level of simulated ischaemia-induced necrosis in the cerebellar cell samples (data not shown). However, samples treated with 7.5 µg/mL of PC AqEP and 10 µg/mL of PC Pg-AqEP decreased the number of necrotic cells by 8.3% and 14%, respectively, compared with the simulated ischaemia-treated samples. The level of necrosis in samples after hypoxia plus DOG treatment was further decreased by application of AqEP and Pg-AqEP at higher concentrations and reached 7.7% with 60 μg/mL of PC AqEP, and 5.9% with 60 μg/mL of PC Pg-AqEP, respectively (Figure 2c,d,g). Differently from AqEP and Pg-AqEP, application of EEP at a small 2 μg/mL of PC concentration significantly decreased the number of necrotic PI-positive nuclei in the hypoxia + DOG-treated samples to 10% (Figure 2e,g). However, application of EEP at a higher concentration (4 μg/mL of PC) did not increase the viability of the hypoxia + DOG-treated cells; the average number of necrotic nuclei in the samples was 16.5%. An EEP at 6 μg/mL of PC did not significantly change the level of necrosis in the hypoxia–DOG-treated cerebellar cell samples (Figure 2f,g).

The effect of different propolis extracts on primary cerebellar cell viability was also evaluated by the level of LDH released into the cell culture medium during the simulated ischaemia treatment. The average level of LDH in the cell culture medium, expressed as a percentage of the total cell LDH in the samples, increased by 28% after the hypoxia + DOG treatment compared to the untreated control (Figure 2h). Addition of 7.5 µg/mL of PC AqEP or 10 µg/mL of PC Pg-AqEP to the cell culture medium did not significantly change the level of LDH induced by simulated ischaemia. Increasing the concentration of AqEP to 15 µg/mL of PC and concentration of Pg-AqEP to 20 µg/mL of PC significantly prevented simulated ischaemia-induced LDH release; the level of LDH was 4.5% lower with 15 µg/mL of PC AqEP and 7% lower with 20 µg/mL of PC Pg AqEP compared to the hypoxia–DOG-treated samples without propolis extracts. A further increase in concentration of both AqEP and Pg AqEP prevented hypoxia–DOG-induced LDH release even more efficiently and reached the level of 7.4% and 3.2% of total cellular LDH content after treatment with 60 µg/mL of PC of AqEP and Pg-AqEP, respectively. However, note that the level of LDH release still remained larger than in normoxic samples, and higher than 60 µg/mL of PC concentrations of the extracts did not further decrease the extent of the simulated ischaemia-induced LDH release (data not shown). Similarly, as in the double nuclear fluorescent viability staining experiments, EEP efficiently prevented simulated ischaemia-induced LDH release, only applied at very small concentrations. A total of 2 µg/mL of PC EEP decreased the average LDH level to 5.8%, and 2 µg/mL of PC EEP to 15.9% of total cellular LDH content. Treatment with 6 µg/mL of PC EEP did not significantly change the simulated ischaemia-induced release of LDH.

There were no significant effects on the number of PI-positive nuclei or LDH release found after propolis extract treatments in normoxia with or without DOG, or in hypoxia without DOG (data not shown). There also were no influence on the propolis extract solvents applied at the same concentrations as the extracts in all normoxic and hypoxic conditions (data not shown).

An increase in number of both PI-positive nuclei and extracellular LDH concentration confirms necrotic cell death induction by simulated ischaemia. There were some pyknotic nuclei observed in the simulated ischaemia-treated cultures after double nuclear viability staining; however, most of them were PI-positive (Figure S1a). This allows to assume that, most likely, apoptosis was initiated by simulated ischaemia, but the cells have switched to necrosis at some later point. Assessment of activity of effector caspase-3, one of the main downstream executors of apoptosis [28], revealed that the enzyme was not significantly more active in the simulated ischaemia-treated samples compared to the normoxic control (Figure S1b). Thus, apoptosis intensity after simulated ischaemia was negligible compared to the level of necrosis.

Summarising, both the Hoechst and PI viability staining and LDH release assays indicate that AqEP and Pg-AqEP protect hypoxia + DOG-treated cerebellar cells from necrotic death in the concentration ranges from 15 µg/mL of PC (AqEP) and 20 µg/mL of PC (Pg-AqEP) to 60 µg/mL of PC. EEP protects cerebellar cells at concentrations ranging from 2 to 4 µg/mL of PC, and in contrary to both hydrophilic propolis extracts, the protection is more efficient at lower concentrations compared to the higher ones.

**Figure 3:**
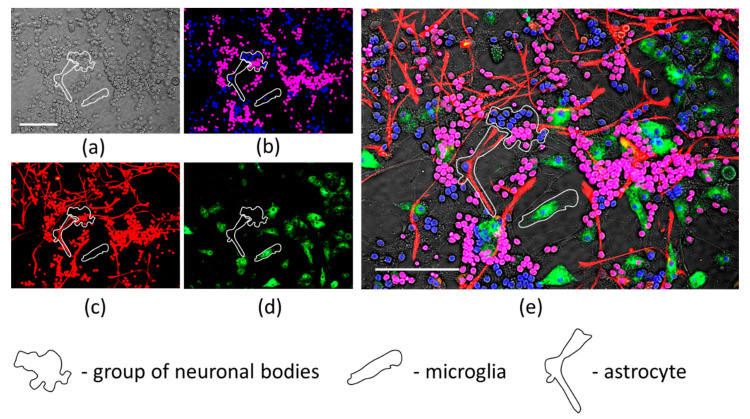
The identification of cells with necrotic nuclei in cell cultures after simultaneous hypoxia and DOG treatment. All panels represent the same microscopic field visualized under different filters or conditions. (**a**)—brightfield phase contrast image; (**b**)—Hoechst- and PI-positive nuclei under DAPI filter; (**c**)—both PI-positive nuclei and GFAP-positive astrocytes under the TxRed filter set; (**d**)—microglia visualized under the FITC filter set; (**e**)—merged image. Typical representatives of each cell type are indicated by white shapes, as explained in the legend. Scale bar is 100 μm.

Next in the study, we have identified the type of cells most vulnerable to hypoxia + DOG-induced necrosis. This was performed by double nuclear staining of cells with Hoechst and PI, and subsequent immunostaining for cell type-specific markers. Astrocytes were visualised by glial fibrillary acidic protein (GFAP) and microglia—by staining with isolectin IB4 from *Griffonia simplificolia*. Neuronal bodies and processes were visualised by brightfield microscopy under a phase contrast objective, and the dead vs. viable cell types were identified according to nuclear co-localisation with the cell bodies (Figure 3). It was identified that 97 ± 3% of the necrotic cells were granule neurons, and only a very small portion of PI-positive nuclei were found in astrocytes or microglia. This finding allows to assume that hypoxia + DOG induce neuronal cell death in mixed neuronal-glial cerebellar cell cultures, and propolis extracts can protect neurons from hypoxia + DOG-induced damage.

### 3.2. The Effect of Propolis Extracts on Hypoxia-Induced Mitochondrial Superoxide Production

In our previous research we have found that propolis extracts can modulate mitochondrial functions and mitochondrial superoxide production in rat astrocytoma cell line C6. Thus, in the present study we have evaluated if the protective effect in 24 h hypoxia-treated cerebellar cell cultures can be related to changes in mitochondrial superoxide generation and mitochondrial function.

**Figure 4:**
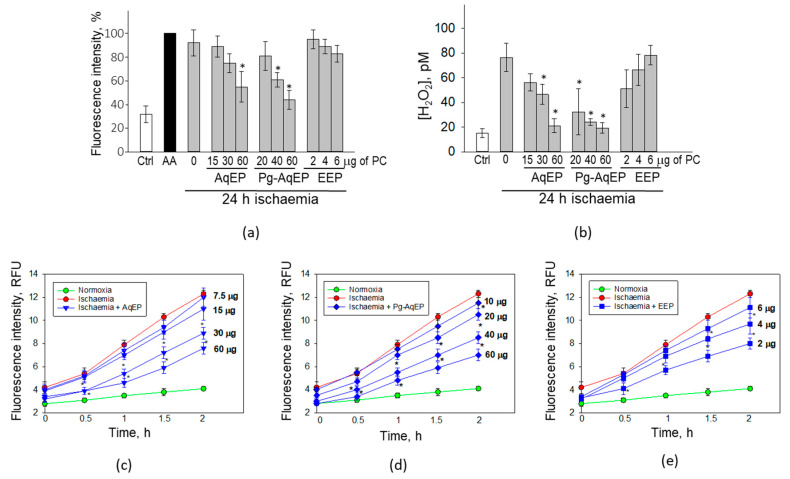
Reactive oxygen species (ROS) production in hypoxia-treated cerebellar neuronal-glial cells, with and without propolis extracts. (**a**)—intramitochondrial superoxide assessed as MitoSOX fluorescence (30 min treatment with 100 μM Antimycin A (AA) was applied as positive control); (**b**)—extracellular level of hydrogen peroxide; (**c**–**e**)—intracellular ROS level measured by DCFDA assay after ischaemia with and without AqEP (**c**), Pg-AqEP (**d**) and EEP (**e**). Concentrations of the extracts are indicated next to the curves. In (**a**), the data are expressed as the percentage of fluorescence with AA. All data are presented as the average ± SE of 3 experimental repeats performed in triplicate. *—statistically significant difference compared with the simulated ischaemia samples (0), *p* < 0.05; ischaemia samples without the extracts were significantly different compared to the normoxic control at all time points, *p* < 0.001.

After treatment with 24 h hypoxia, generation of mitochondrial superoxide increased by 60%, reaching a level similar to that induced by mitochondrial respiratory complex III inhibitor antimycin A (Figure 4a). Treatments with 15–30 µg/mL of PC AqEP, 20 µg/mL of PC Pg AqEP and 2–6 µg/mL of PC EEP did not significantly affect the level of hypoxia-induced mitochondrial superoxide production. However, 60 µg/mL of PC AqEP and 40–60 µg/mL of PC Pg-AqEP significantly decreased the level of mitochondrial superoxide evaluated after hypoxia treatment. The superoxide level found in mitochondria treated with 60 µg/mL of PC AqEP was by 37% lower, and after treatment with 40–60 µg/mL of PC Pg-AqEP, 31–48% lower compared to the hypoxic control.

Superoxide is converted to hydrogen peroxide (H_2_O_2_) by mitochondrial superoxide dismutase, and H_2_O_2_ can easily diffuse through the mitochondrial and plasma membranes to the extracellular medium. If superoxide generation is increased in cells after simulated ischaemia, it should also be reflected in production of extracellular H_2_O_2_. Indeed, the level of extracellular H_2_O_2_ after simulated ischaemia was 5 times higher compared to the normoxic samples (Figure 4b). In the medium of the samples treated by simulated ischaemia in the presence of 30–60 µg/mL of PC AqEP and 20–60 µg/mL of PC Pg-AqEP, the concentration of H_2_O_2_ was significantly lower than in the simulated ischaemia samples without the propolis extracts. The decrease in H_2_O_2_ concentration by the extracts was dose dependent, and the levels of H_2_O_2_ after simulated ischaemia plus 60 µg/mL of PC AqEP and simulated ischaemia plus 60 µg/mL of PC Pg-AqEP treatments were close to those levels in the normoxic control (21.1 ± 5.6 pM, 19.4 ± 4.3 pM and 15.1 ± 3.4 pM, respectively). There were no statistically significant changes in H_2_O_2_ concentration in the EEP-treated ischaemic samples compared to the ischaemic control. Overall, the propolis extracts were similarly efficient in both preventing simulated ischaemia-induced mitochondrial superoxide formation and extracellular hydrogen peroxide generation.

Evaluation of intracellular hydroxyl, peroxyl and other ROS by DCFDA assay revealed a significantly faster increase in the species inside the simulated ischaemia-treated cells compared to the samples that were kept in normoxic conditions (Figure 4c–e). After 2 h of incubation, the level of DCF fluorescence in the simulated ischaemia-treated samples was three times higher compared to the normoxic control. AqEP up to 15 µg/mL of PC did not significantly affect the level of ROS production (Figure 4c). In the samples incubated under ischemic conditions together with 30–60 µg/mL PC AqEP, the ROS level was significantly lower compared to the ischaemic samples at all evaluation time points. After 2 h of monitoring with 30–60 µg/mL PC, the DCF fluorescence intensity was 35–52% lower than in the ischaemic samples. Similar ROS generation kinetics was observed in the ischaemic samples treated with Pg-AqEP. The 10–20 µg/mL PC Pg-AqEP treatment tended to decrease ROS production, but both the 30 and 60 µg/mL PC Pg-AqEP significantly lowered ROS generation by 38% and 57%, respectively (Figure 4d). In the EEP-treated ischaemic samples, only the concentration of 2–4 µg/mL PC was efficient in decreasing the level of the intracellular ROS production rate (Figure 4e). After 2 h of monitoring, in the 2–4 µg/mL PC EEP-treated ischaemic samples, the DCF fluorescence level was by 35–45% lower compared to the ischaemic samples without the extracts.

### 3.3. The Effect of Propolis Extracts on Mitochondrial Function in Cerebellar Cells Treated by Hypoxia

For the mitochondrial function evaluation experiments, the most viability-protecting concentrations of propolis were selected: 60 µg/mL of PC for AqEP and Pg-AqEP, and 2 µg/mL of PC for EEP.

**Figure 5:**
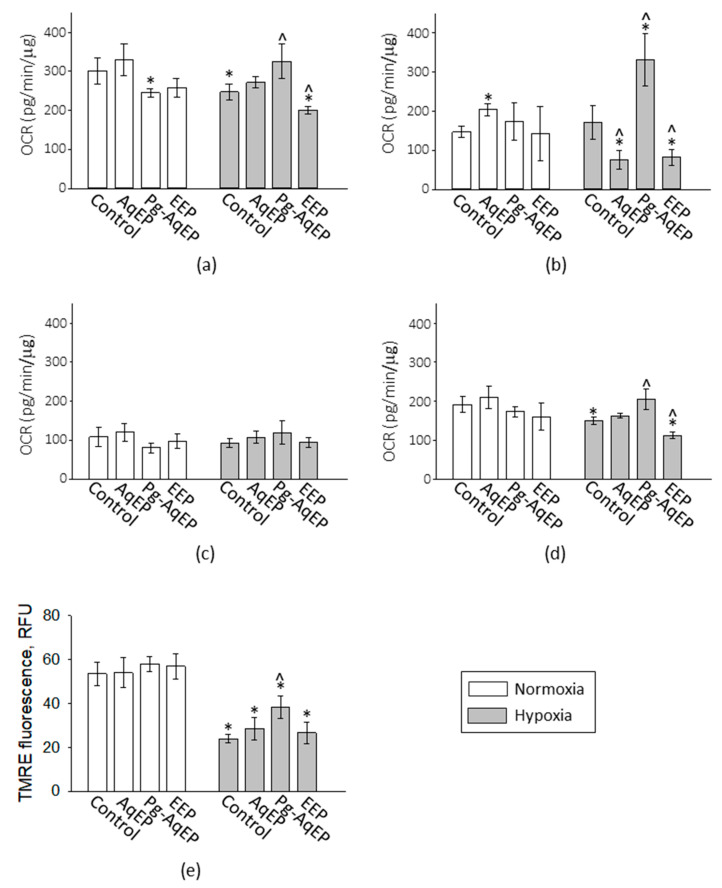
Mitochondrial function in hypoxia-treated cerebellar neuronal-glial cells with and without propolis extracts. The bioenergetics of the cerebellar cells were assessed by Seahorse Flux Analyzer using Cell Mito Stress Kit, and the summary data calculated from the curves presented in Supplementary Figure S1, are shown in this figure. Panel (**a**) shows the basal mitochondrial respiration rate expressed as the oxygen consumption rate (OCR); panel (**b**) is the maximal mitochondrial respiratory capacity, (**c**) is the OCR induced by the proton leak; and (**d**) the OCR that is coupled with phosphorylation and used for ATP production. (**e**) The mitochondrial membrane potential evaluated according to TMRE fluorescence. The data are normalized for total cellular protein and presented as the average ± SE of 3 experimental repeats performed in triplicate. *—statistically significant difference compared to the normoxic control, *p* < 0.05; ^—statistically significant difference compared to the hypoxic control, *p* < 0.05.

Assessment of the basal mitochondrial respiration rate in normoxia revealed no significant difference between the control and AqEP-treated samples, nor between the control and EEP-treated samples; however, the Pg-AqEP treatment decreased the mitochondrial basal respiration rate by 19% (Figure 5a, white bars). There was no significant change in the proton leak and ATP synthesis-coupled respiration in the normoxic samples (white bars in Figure 5c,d). However, the spare respiratory capacity that was activated by mitochondrial uncoupler CCCP was increased by AqEP (by 38%) compared to the control (Figure 5b).

After the 24-h hypoxia treatment, mitochondrial basal respiration and ATP synthesis-coupled mitochondrial respiration was significantly decreased. The decrease in basal respiration was by 18%, and in ATP-related respiration by 19% (Figure 5a,d). Treatment with AqEP and EEP did not prevent hypoxia-induced decrease in both basal and ATP-coupled mitochondrial respiration. Moreover, EEP even decreased the rates by 19% and 27%, respectively, compared to the hypoxic control. EEP also significantly (by 52%) decreased the spare mitochondrial respiratory capacity of the hypoxic cells, and some decrease after treatment with this extract was noticeable also in the proton leak-driven respiration (Figure 5b,c). Spare respiratory capacity in hypoxic samples was also similarly inhibited by AqEP. However, application of Pg-AqEP completely prevented hypoxia-induced loss of the mitochondrial basal and ATP-coupled respiration rate; both rates after treatment with this extract were similar to the level of normoxic control. Moreover, in hypoxic samples, Pg-AqEP significantly increased the spare mitochondrial respiratory capacity. There was no significant effect of both AqEP and Pg-AqEP on proton leak under hypoxic conditions.

Evaluation of the mitochondrial inner membrane potential, Δψ, by TMRE fluorescence assay revealed a significant decrease (more than twice of the value) in Δψ after simulated ischaemia treatment compared to the normoxic samples (Figure 5e). The presence of Pg-AqEP significantly prevented an ischaemia-induced decrease in the mitochondrial membrane potential; the average level of TMRE fluorescence increased by 60% in the presence of 60 µg/mL of PC Pg-AqEP. AqEP and EEP applied in hypoxia did not significantly change the mitochondrial Δψ compared to the hypoxic control. Neither of the extracts had an influence on mitochondrial Δψ in normoxia.

The data about mitochondrial function in hypoxia and the propolis extract-treated samples indicate that the protective activity of Pg-AqEP might be related to an improvement in the mitochondrial respiratory capacity and, subsequently, a decreased level of mitochondrial superoxide production. However, the protective activity of AqEP and EEP could hardly be related to mitochondrial modulation.

### 3.4. The Effect of Propolis Extracts on Hypoxia-Induced Release of Inflammatory Cytokines

**Figure 6:**
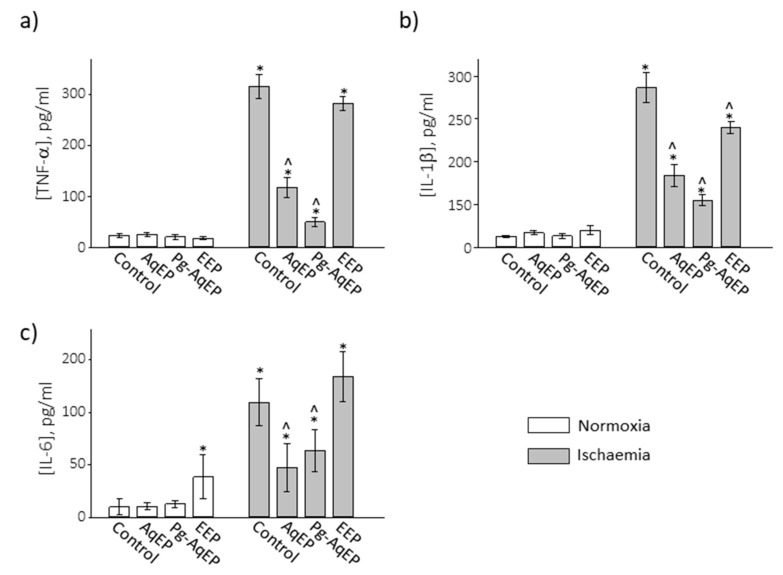
Concentration of proinflammatory cytokines TNF-α (**a**), IL-1β (**b**) and IL-6 (**c**) in a medium of cultured primary cerebellar neuronal-glial cells after simulated ischaemia (hypoxia plus DOG) treatment. The data are presented as the average ± SE of 3 experimental repeats performed in quintuplicate. *—statistically significant difference compared to the normoxic control, *p* < 0.05; ^—statistically significant difference compared to the hypoxic control, *p* < 0.05.

Oxidative stress induced by hypoxia leads to the release of inflammatory cytokines from glial cells and consequent neuronal death [12]. Next in the study, we have investigated whether propolis extracts affect the level of TNF-α, IL-1β and IL-6 released from the cultivated neuronal-glial cells after hypoxia treatment. The same as for the mitochondrial function evaluation experiments, the most viability-protecting concentrations of propolis were selected: 60 µg/mL of PC for AqEP and Pg-AqEP, and 2 µg/mL of PC for EEP. In normoxia, all the examined cytokines were found in concentrations that did not exceed 25 pg/mL, in both the control and propolis extract-treated samples, with the exception of IL-6 after EEP treatment, which was nearly 4 times higher compared to the untreated control (Figure 6). There was no significant change in cytokine level after treatment of DOG in normoxia (data not shown). When the cells were subjected to ischaemia (hypoxia plus DOG), the levels of inflammatory cytokines in the medium were 13, 14 and 11 times higher compared to the normoxic control for TNF-α, IL-1β and IL-6, respectively. When the cell culture medium was supplemented with 60 µg/mL of PC AqEP, the concentration of TNF-α was 2.7 times lower compared to the ischaemic control (Figure 6a). When 60 µg/mL of PC Pg-AqEP was present in the medium, the level of the cytokines was 6.3 times lower and became close to the level in the normoxic samples. However, 2 µg/mL of PC for EEP did not significantly prevent the cells from an ischaemia-induced increase in TNF-α concentration. The similar pattern of protection by propolis extracts was observed for IL-1β (Figure 6b). AqEP and Pg-AqEP lowered the level of the cytokines in the ischaemic samples by 2.2 and 3.4 times. EEP also significantly decreased the IL-1β level, but the protection was much smaller. The hydrophilic extracts AqEP and Pg-AqEP also were effective in decreasing the IL-6 concentration, which was elevated by the ischaemia (Figure 6c). The concentration of IL-6 after treatment with AqEP or Pg-AqEP in ischaemic conditions was by about 2 times smaller than in the ischaemic control samples. EEP applied together with ischaemia did not significantly change the level of IL-6 in the medium. The treatment of cells with hypoxia alone without DOG did not significantly influence the level of investigated inflammatory cytokines (data not shown).

Summarising, both the hydrophilic propolis extracts AqEP or Pg-AqEP significantly (yet not completely) prevented ischaemia-induced inflammatory cytokine TNF-α, IL-1β and IL-6 release from cultured primary cerebellar neuronal-glial cells. However, EEP was protective only against ischaemia-induced IL-1β release, and the protection extent was relatively small.

## 4. Discussion

The human brain comprises only about 2% of the body’s weight, but it receives almost 15% of the cardiac output because of the extremely high demand of oxygen and metabolite exchange [29]. During development of ischaemia, progressive decrease in cerebral oxygen consumption is always followed by a decrease in the level of brain cell functional performance [30]. A further reduction in the blood flow causes critical oxygen and glucose deprivation and brain tissue subsequently undergoes cell death [31]. According to the data of the WHO, the number of individuals suffering death or disability after cerebral ischaemia increases, and the average age of such individuals decreases [32]. Anti-thrombolytics and hypothermia are the only up to date effective strategies in reducing neuronal injury, neurological deficits and mortality rates following cerebral ischaemia, and development of more efficient novel therapies against stroke/cerebral ischaemia are urgently needed [33].

Numerous studies indicate that cell death after cerebral ischaemia is exclusively necrotic in nature [34,35]. However, there is also some evidence that apoptosis might also occur in vitro after hypoxia [36]. Apoptotic cell death pathways have also been implicated in ischemic cerebral injury in in vivo ischaemia models [37]. In our experiments described in the current study, 24 h treatment with hypoxia and DOG caused death in over 30% of primary neuronal and glial cells, and the final stage of the death pathway had necrotic features, such as the lost integrity of the nuclear membrane and release of cellular LDH. The presence of pyknotic nuclei, which is assumed as one of the markers of apoptosis [38] in simulated ischaemia-treated samples, indicate that the apoptotic cell death pathway could be initiated. However, most of the pyknotic nuclei were PI-positive at the time point of evaluation, indicating necrosis as the final cell death pathway (Figure S1a), and the level of active effector caspase-3 after simulated ischaemia was not significantly different from the normoxic control samples (Figure S1b). One of the possible explanations of this could be the insufficient amount of energetic resources to complete apoptosis due to the shortage of oxygen and glycolysis inhibition. Other studies of primary neuronal cell cultures and in vivo neonatal mice also indicate that apoptosis occurs during the reoxygenation period rather than in the hypoxic phase [36,39].

A 24-h treatment under hypoxic condition did not induce a significant increase in cell death. Neuronal necrosis was only detected when hypoxia was applied together with inhibition of glycolysis by DOG. In addition, DOG also did not induce a significant impact on the level of cell death in normoxia. This suggests that the synergy of the oxygen and glucose deprivation pathways are essential for ischaemia-induced death. The vast majority of necrotic cells found in simulated ischaemia-treated neuronal-glial cell cultures were cerebellar granule neurons. A similar finding was described in primary cerebral culture, showing that oxygen–glucose deprivation (but not hypoxia alone) promotes ROS, loss of mitochondrial complex I activity and necrosis in neurons, but not in astrocytes [40]. Thus, our study confirms the reports of other authors that neurons are more sensitive to ischaemic damage compared to other cell types, and that necrosis is the dominating cell form during ischaemia, most likely because of the insufficient energetic resources to perform more complicated death pathways.

Previously, we have found that the differently prepared propolis extracts AqEP, Pg-AqEP and EEP were efficient scavengers of extracellular, intracellular and mitochondrial ROS in the astrocytoma C6 cell line [24]. In addition, the extracts revealed significant stimulation of mitochondrial respiration by 32 µg/mL of PC and higher Pg-AqEP. In this study, we continued to explore these biological properties by testing them on primary brain cells in normoxic, hypoxic (2% oxygen) and ischaemia-mimicking (2% oxygen plus 10 mM DOG) conditions. Each of the investigated extracts were protective against such hypoxia + DOG-induced cytotoxicity; however, both the efficient concentration and protection level were not the same: 60 µg/mL of PC Pg-AqEP revealed the highest level of protection—only 6% of necrotic nuclei and 4% of total LDH content were released. Slightly lower yet similar level of protection was achieved by 60 µg/mL of PC AqEP (8% of necrotic nuclei and 7.5% of total LDH content). EEP was protective only when applied in small concentrations of 2 µg/mL of PC (decreased the number of necrotic nuclei to 10% and LDH release to 6%). However, a further increase in EEP concentration up to 6 µg/mL of PC completely eliminated the protection against simulated ischaemia-induced death. This indicates that the EEP content and activity is different from both AqEP and Pg-AqEP.

There are very little literature data available about the effect of propolis on brain cell viability after ischaemia. Shimazawa and co-authors investigated the effect of aqueous and ethanolic extracts of Brazilian green propolis in vitro (PC12 cells, 0.4–40 µg/mL) and an aqueous extract in vivo (mice, 30 or 100 mg/kg) and found that propolis significantly inhibited ischaemia-induced neurotoxicity [21]. Abdel-Rahman and colleagues revealed that in ischaemia/reperfusion-treated groups, propolis (applied at 50 and 100 mg/kg) significantly reduced neurodegeneration and histological alterations in the brain tissues [22]. Bazmandegan and co-authors investigated the effect of water-extracted brown propolis (100 and 200 mg/kg) against cerebral ischaemia-induced oxidative injury in a mouse model of stroke and demonstrated that treatment resulted in significant restoration of antioxidant enzyme (superoxide dismutase and glutathione peroxidase) activity and a subsequent decrease in the infarct volume compared to the control group. The authors have concluded that the neuroprotective activity of the propolis extracts is related to the stimulation of an antioxidant mechanism, which seems to be mediated by the endogenous antioxidant system [41]. We have also previously revealed that AqEP, Pg-AqEP and EEP at 5–15 µg/mL of PC efficiently neutralise hydrogen peroxide and scavenge/prevent production of intracellular ROS as well as mitochondrial superoxide [24]; however, the data were obtained under normoxic conditions. In this study, we confirmed the mitochondrial superoxide-preventing properties for 60 µg/mL of PC AqEP and 40–60 µg/mL of PC Pg-AqEP in hypoxia. These findings allow to suggest that the cytoprotective efficiency of AqEP and Pg-AqEP might be exerted by modulation of the mitochondrial superoxide levels. However, there were no significant protection against hypoxia-induced mitochondrial superoxide provided by 2–6 µg/mL of PC EEP. A similar pattern of the extract efficiency was also observed for extracellular H_2_O_2_ and intracellular ROS generation measured immediately after ischaemia. Note that the extracts were not present in the medium during the ROS generation assessment; thus, the effect was not due to direct ROS scavenging. Treatment with 30–60 µg/mL of PC AqEP and Pg-AqEP and 2 µg/mL of PC EEP prevented ischaemia-induced ROS generation in mitochondria and cells during the initial 0.5–2 h of reoxygenation (the evaluation of ROS was performed in normoxic conditions).

Most likely, the amounts of biologically active compounds capable to interfere with the superoxide were too low, because at higher concentrations (10–15 µg/mL of PC) EEP was very efficient in decreasing superoxide production by C6 cell mitochondria [24]. Thus, cytoprotection of 2–4 µg/mL of PC EEP from simulated ischaemia-induced damage was not related to the change in mitochondrial superoxide levels. Wozniak and co-authors (2019) investigated the antioxidant properties of EEP by Fe^3+^-reducing power and ferrous ion (Fe^2+^)-chelating activity assays and haemolytic activity by applying 10–100 µg/mL of PC, and found that the extracts were 16–18% protective against AAPH-induced haemolysis [42]. Oses and colleagues discovered that half the maximal inhibitory concentration (IC50) of the antioxidant activity against the superoxide anion radical was 190 µg/mL for EEP [43]. Thus, the efficient antioxidant concentrations of the propolis extracts found in our study are similar to those revealed by other authors in various biological systems.

Ischaemia-induced mitochondrial superoxide production might cause mitochondrial damage and dysfunction. To test if this might be the case in our simulated ischaemia model, we have examined mitochondrial function by a Seahorse XFp Analyser. In normoxia, there were no large-extent changes in mitochondrial function induced by the different propolis extracts. However, in hypoxia, different extracts exerted distinct activities on the mitochondrial functional components. EEP further contributed to inhibition of mitochondrial respiration induced by hypoxia. AqEP also decreased the mitochondrial spare respiratory capacity in hypoxic samples, although this was not reflected in the basal respiration rate. Thus, protection of neuronal cells from ischaemia-induced necrosis by these extracts could not be related to enhancement of mitochondrial function. On the other hand, mild inhibition of mitochondrial respiration could under some circumstances be cytoprotective. For example, inhibition of mitochondrial respiratory complex I activity by metformin or rotenone is demonstrated to prevent ischaemia-induced opening of mitochondrial permeability transition pore and subsequent cell death [44,45]. The relationship between complex I and mitochondrial permeability transition was also suggested to mediate brain injury in hypoxia/hyperoxia-reperfusion in 10-day-old mice [46]. Thus, it might be that the protective effect of both AqEP and EEP is related to the slow-down of complex I activity and a decrease in the probability of mitochondrial permeability transition; however, to confirm this hypothesis, further studies are required.

The effect of Pg-AqEp on mitochondrial function in hypoxia was in high contrast to that of the other extracts; this was the only extract that completely prevented hypoxia-induced inhibition of the mitochondrial basal respiration and phosphorylation system, protected from mitochondrial inner membrane potential loss induced by ischaemia and, surprisingly, strongly stimulated the maximal mitochondrial respiratory capacity, revealed in the presence of the uncoupler CCCP. Although in the previous study on C6 cells it was found that Pg-AgEP has uncoupling properties, this was not the case in the present study, as the level of proton leak-driven respiration was not stimulated by this extract. The presence of polyethylene glycol is likely to increase the penetration of the propolis active compounds into the mitochondrial membranes, and this might facilitate electron transfer between the mitochondrial respiratory complexes. Plant-derived phenolic compounds are known to modulate mitochondrial function, dynamics and maintenance [47], mostly by mild uncoupling and interaction with mitochondrial respiratory chain complexes [48]. However, a recent study of the impact of a pre-fermented mixture of polyphenols (Rechtsregulat^®^) has revealed stimulation of the mitochondrial oxidative phosphorylation system via stimulation of the respiratory chain complexes I, II and IV [49]. The composition analysis of the preparation had some similarities with the propolis extracts, including the presence of phenolic acids, such as p-coumaric acid. These data allow to suggest that propolis extract compounds might also have a similar effect on the mitochondrial respiratory chain components, and specific preparation of Pg-AqEP might potentiate them for mitochondria-stimulating efficiency. Altogether, this lays the basis for further studies dedicated to the exploration of the effect of Pg-AqEP on the components of the mitochondrial respiratory chain and phosphorylation system in normal and ischaemic conditions.

Both the hydrophilic extracts AqEP and Pg-AqEP showed significant protection from the ischaemia-induced increase in inflammatory cytokine level. The anti-inflammatory properties of propolis have been described by many authors under different experimental conditions [50,51]. More specifically, Wu and co-authors have found that Brazilian green propolis (50 μg/mL) significantly prevents the hypoxia-induced release of proinflammatory cytokines IL-1β, TNF-α and IL-6; mitochondrial ROS generation; activation of nuclear factor-κB (NF-κB), and protects MG6 mouse microglial cells from hypoxia-induced death [52]. Moreover, the anti-inflammatory response in hypoxia-exposed mice was also demonstrated in vivo after systemic 7-day administration of propolis. Thus, the cytoprotective activity of AqEP and Pg-AqEP could likely be explained by inhibition of mitochondrial ROS and subsequent prevention of cytokine production/release via NF-κB in both microglia and astrocytes. Pg-AqEP more efficiently prevented ischaemia-induced IL-1β and TNF-α release compared to AqEP, and this correlated with more pronounced protection of mitochondrial functions by the extract. Such evidence allows to suggest Pg-AqEP as a potential neuroprotective preparation in the case of cerebral ischaemia.

## 5. Conclusions

All the investigated propolis extracts protect cultivated primary cerebellar neuronal-glial cells from simulated ischaemia (hypoxia plus DOG)-induced necrosis, which primarily affects neuronal cells. The AqEP and Pg-AqEP protective concentration range is 15–60 µg/mL of PC, and the protective efficiency increases together with the concentration. EEP protects cerebellar cells at concentrations ranging from 2 to 4 µg/mL of PC, and in contrast to both the hydrophilic propolis extracts, the protection is more efficient at lower concentrations compared to the higher ones.

Both AqEP and Pg-AqEP possess the ability to prevent ischaemia-induced production of extracellular and intracellular ROS and mitochondrial superoxide, indicating this might be the pathway of cytoprotection from ischaemia-induced damage. However, EEP does not possess mitochondrial superoxide- and extracellular H_2_O_2_-preventing activity; thus, EEP protects ischaemia-affected cells in a way not related to mitochondrial superoxide.

Pg-AqEP prevents a hypoxia-induced decrease in the mitochondrial respiratory chain, phosphorylation activity and inner membrane potential loss, and stimulates mitochondrial respiratory capacity. Such effects might explain the lower mitochondrial superoxide level and neuronal survival after ischaemia. AqEP has no effect on mitochondrial functions, but EEP has inhibitory activity; thus, further studies are needed to find the mechanisms by which these extracts protect brain cells from ischaemia-induced death.

Pg-AqEP (60 µg/mL of PC) most potently prevents ischaemia-induced release of inflammatory cytokines, and AqEP (60 µg/mL of PC) follows with a similar efficiency. EEP (2 µg/mL of PC) slightly prevents the release of IL-6, but does not prevent increases in IL-1β and TNF-α.

Pg-AqEP prevents ischaemia-induced cell death via decreasing ROS, protecting mitochondria and preventing inflammation more efficiently compared to AqEP and EEP.

## Supplementary Materials

The following are available online at https://www.mdpi.com/2076-3921/9/12/1262/s1, Figure S1: The effect of hypoxia and deoxyglucose (DOG) on the number of chromatin-condensed nuclei (**a**) and caspase-3 activity level (**b**) in mixed neuronal-glial cultures; Figure S2: Average values of the mitochondrial oxygen consumption curves obtained by the Seahorse XFp Analyser.
