# Supplementary material for: The Role of Mitochondria in Brain Cell Protection from Ischaemia by Differently Prepared Propolis Extracts
Source: Antioxidants (Basel). 2020 Dec 12;9(12):1262. doi: 10.3390/antiox9121262 (PMC7763930; doi:10.3390/antiox9121262)
Supplement: Supplementary file 1 [file antioxidants-09-01262-s001.zip › Figure S1.pdf]

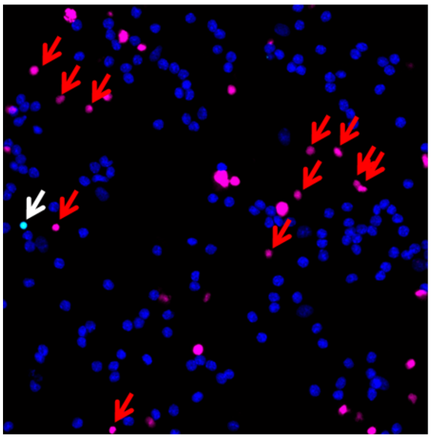

(a)

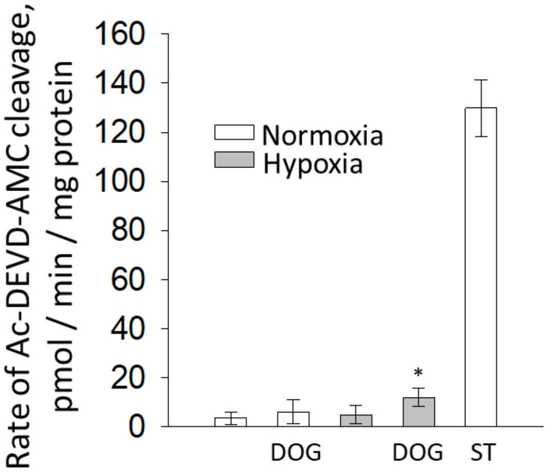

(b)

Figure S1. The effect of hypoxia and deoxyglucose (DOG) on number of chromatin-condensed nuclei (a) and caspase-3 activity level (b) in mixed neuronal-glial cultures. White arrow in (a) indicate a chromatin-condensed nucleus of a viable cell which is typical for apoptosis. Red arrows indicate condensed yet necrotic (propidium iodide-positive) nuclei. ST – 3 hour treatment with 100 nM staurosporine. * - significant difference compared to normoxic control, $p < 0.05$.
