# Supplementary material for: The Role of Mitochondria in Brain Cell Protection from Ischaemia by Differently Prepared Propolis Extracts
Source: Antioxidants (Basel). 2020 Dec 12;9(12):1262. doi: 10.3390/antiox9121262 (PMC7763930; doi:10.3390/antiox9121262)
Supplement: Supplementary file 1 [file antioxidants-09-01262-s001.zip › Figure S2.pdf]

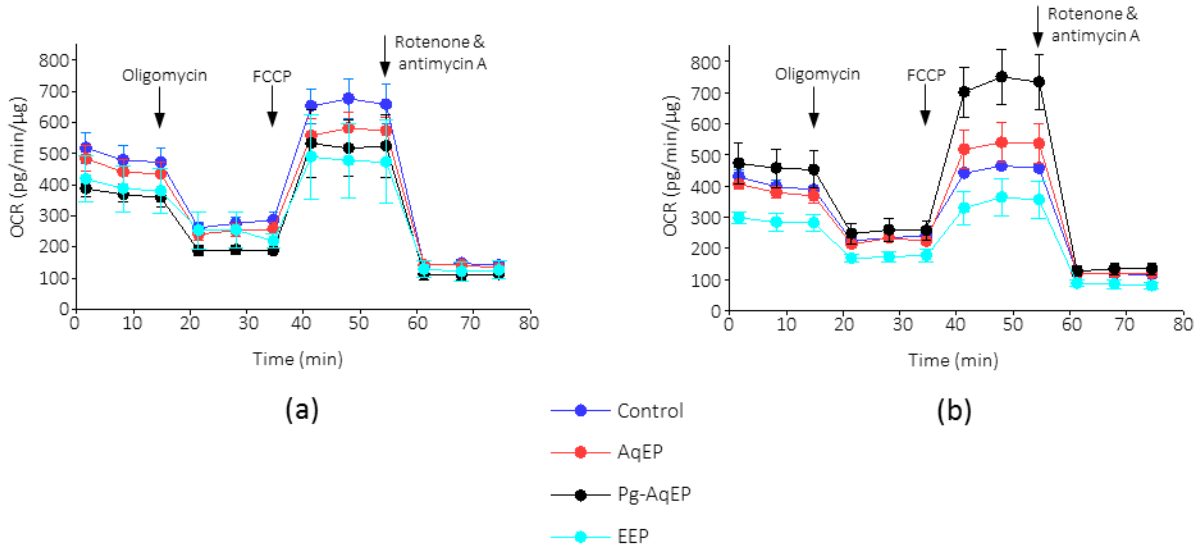

Figure S2 Average values of mitochondrial oxygen consumption curves obtained by Seahorse XFp Analyser. (a) – cells were treated with 60 µg/ml of PC AqEP, or with Pg-AqEP, or 2 µg/ml of PC for EEP for 24 hours in normoxic conditions; (b) – after the same treatment in hypoxic conditions (2% oxygen). The data are presented as averages of 3 experiments ± standard deviation.
